# *CYP1A2* genotype-dependent effects of smoking on mirtazapine serum concentrations

**DOI:** 10.1177/02698811251337387

**Published:** 2025-05-12

**Authors:** Maike Scherf-Clavel, Heike Weber, Carolin Weiß, Catherina Klüpfel, Saskia Stonawski, Leif Hommers, Stefan Unterecker, Katharina Domschke, Andreas Menke, Sarah Kittel-Schneider, Sebastian Walther, Jürgen Deckert, Angelika Erhardt-Lehmann

**Affiliations:** 1 Department of Psychiatry, Psychosomatics, and Psychotherapy, Center of Mental Health, University Hospital of Würzburg, Würzburg, Germany; 2 Interdisciplinary Center for Clinical Research, University Hospital of Würzburg, Würzburg, Germany; 3 Department of Clinical Research and Epidemiology, Comprehensive Heart Failure Center, University Hospital of Würzburg, Würzburg, Germany; 4 Faculty of Medicine, Department of Psychiatry and Psychotherapy, Medical Center – University of Freiburg, University of Freiburg, Freiburg, Germany; 5 Department of Psychosomatic Medicine, and Psychotherapy, Medical Park Chiemseeblick, Bernau am Chiemsee, Germany; 6 Department of Psychiatry and Psychotherapy, University Hospital, Ludwig Maximilian University of Munich, Munich, Germany; 7 Department of Psychiatry and Neurobehavioural Science, University College Cork, Cork, Ireland; 8 APC Microbiome, University College Cork, Cork Ireland; 9 Max Planck Institute of Psychiatry, Munich, Germany

**Keywords:** Mirtazapine, pharmacogenetics, serum concentration, *CYP1A2*, smoking cigarettes

## Abstract

**Introduction:**

Psychopharmacotherapy with mirtazapine is commonplace. Lower serum concentrations of mirtazapine were reported in smokers due to *CYP1A2* induction. However, no previous study that investigated *CYP1A2* genetics and mirtazapine treatment considered *CYP1A2-*inducing parameters.

**Aim:**

We aimed to investigate the association of *CYP1A2* variants, mirtazapine serum concentration, and treatment outcome, considering the smoking status of the patients.

**Methods:**

Two depression cohorts were investigated for the association between serum concentration and treatment response of mirtazapine and *CYP1A2*-163C>A (rs762551) and -3860G>A (rs2069514) genotype groups, also considering smoking status, sex, and age of the patients. In total, 124 patients (82 non-smokers and 42 smokers) were eligible for the analyses.

**Results:**

Dose-corrected serum concentration (CD) of mirtazapine was associated with smoking status, sex, and age, with lower CD in smokers, females, and older patients. Considering non-smokers and genotype-grouped smokers, CD of mirtazapine in *CYP1A2* normal metabolizer smokers (*N* = 6) did not differ from CD of non-smokers. By contrast, smokers carrying the CYP1A2*1A/*1F and *1F/*1F genotype groups showed 34.4% and 33.4% lower mirtazapine CD compared to non-smokers.

**Discussion:**

As yet, for clinical practice, it may be more relevant to focus on smoking status than on the *CYP1A2* gene variants. Considering the relevant impact of smoking on the mirtazapine CD, physicians should monitor an increase in side effects due to the expected increase in mirtazapine serum concentrations. In these cases, measurement of mirtazapine CD and/or subsequent dosage reduction is recommended. The clinical relevance of *CYP1A2* genotyping prior to treatment with drugs metabolized by *CYP1A2* needs further investigation.

## Introduction

The association between smoking cigarettes and depression is well known (Brown et al., 1996; Chaiton et al., 2009); over 30% of patients with current major depressive disorders (MDDs) and nearly 60% of patients with a lifetime history of depression are smokers (Ziedonis et al., 2008). Moreover, smoking is significantly associated with the risk of suffering from depression (Breslau et al., 1991).

In addition, smoking cigarettes has an impact on the treatment of MDD, as, for example, the polycyclic aromatic hydrocarbons in cigarette smoke induce Cytochrom P450 1A2 (*CYP1A2*; Kroon, 2007). For smokers, a 1.55-fold higher *CYP1A2* activity was reported compared to non-smokers (*p* < 0.0001) (Dobrinas et al., 2011). In addition, the number of cigarettes was associated with *CYP1A2* activity (Dobrinas et al., 2011). Consequently, in smokers, serum concentrations of antidepressants metabolized by *CYP1A2* may be lower than expected due to the increased metabolism (Scherf-Clavel et al., 2019), possibly resulting in insufficient serum concentrations below the therapeutic reference range (TRR; Hiemke et al., 2018). Besides smoking cigarettes, *CYP1A2* activity is affected by other non-genetic factors, for example, drugs that inhibit or induce *CYP1A2* (e.g., fluvoxamine (inhibitor), carbamazepine (inducer)) (Flockhart et al., 2021). Furthermore, *CYP1A2* activity is affected by variations in the *CYP1A2* gene, particularly the 163C>A (rs762551) single-nucleotide polymorphism (SNP) (Royal Dutch Pharmacist’s Association, 2021). *CYP1A2* activity was reported to be increased in association with the CYP1A2*1F genotype (rs762551), that is, homozygosity or heterozygosity for the A allele (Dobrinas et al., 2011; Royal Dutch Pharmacist’s Association, 2021). Moreover, it was suggested that this CYP1A2*1F genotype confers a higher inducibility through smoking compared to other genotypes, with a 1.6-fold higher metabolic activity in smokers carrying the *1F/*1F variant compared to other genotypes (Sachse et al., 1999). CYP1A2*1A is considered as reference haplotype (rs762551; C allele).

Inducibility of *1F is supported by a study on olanzapine (Laika et al., 2010), primarily metabolized by *CYP1A2* (Hiemke et al., 2018). For olanzapine, a significant association between the CYP1A2*1F genotype and the serum concentrations was reported, with lower concentrations in CYP1A2*1F/*1F carriers compared to CYP1A2*1A carriers (rs762551; C allele) (Laika et al., 2010). Moreover, including only patients with *CYP1A2* inducers (tobacco smoke and carbamazepine), 22% of lower serum concentrations of olanzapine in CYP1A2*1F/*1F carriers were reported compared to CYP1A2*1A carriers (Laika et al., 2010). Clozapine is another drug primarily metabolized by CYP1A2 (Hiemke et al., 2018). Also, for clozapine, lower serum concentrations were reported in smokers who were CYP1A2*1F/*1F carriers (Sangüesa et al., 2022). By contrast, in another study, serum concentrations of clozapine did not differ in patients with different *CYP1A2* genotypes; however, the study just distinguished between CYP1A2*1A/*1A and CYP1A2*1F carriers (*1A/*1F and *1F/*1F) (van der Weide et al., 2003).

Mirtazapine is one of the most prescribed antidepressants in Germany in 2022 (Seifert et al., 2022). Studies investigating *CYP1A2* genetics in association with mirtazapine treatment did not report an association between mirtazapine serum concentration and *CYP1A2* variants (Jaquenoud Sirot et al., 2012; Scherf-Clavel et al., 2022). Nevertheless, no study so far has focused on mirtazapine serum concentration and *CYP1A2* gene variants, taking into account *CYP1A2*-inducing parameters, such as smoking.

Therefore, the present analysis aimed to investigate the association of *CYP1A2* variants and mirtazapine treatment, considering the smoking status of the patient. We hypothesized that in smokers, who are CYP1A2*F carriers, the dose-corrected serum concentration (CD) of mirtazapine was lower compared to smoking patients, who are carriers of other genotypes. Moreover, we hypothesized that CDs in non-smoking patients were not different across the genotypes.

## Methods

### Participants

#### Wuerzburg sample

The Wuerzburg sample was collected within the GEParD (Genetics and Epigenetics of Pharmaco- and Psychotherapy in acute and recurrent Depression, recruitment 2012–2020) study (Lichter et al., 2023). A total of 346 patients (mean age 45.6 ± 15.3 years (range 18–80); 58% female) admitted to the Department of Psychiatry, Psychosomatics, and Psychotherapy of the University Hospital of Wuerzburg, due to a depressive episode (single major depressive episode, recurrent depression, or bipolar depression), were included in the study within the first 5 days after admission and weekly measurements were performed for up to 7 weeks (Lichter et al., 2023). Diagnosis of depressive episodes was ascertained by specialized staff according to the Diagnostic and Statistical Manual of Mental Disorders (DSM)-IV criteria. Symptom severity and effectiveness of the therapy were assessed by the Hamilton Depression Rating Scale (HAMD)-21 (mean ± standard deviation (SD) at baseline: 20.6 ± 6.0). Exclusion criteria were inability to give written informed consent, presence of a depressive disorder due to substance use disorder, severe neurological or general medical conditions, diagnosis of schizophrenia/psychosis, and systemic medication with glucocorticoids (Lichter et al., 2023). All patients were treated independently of study participation (naturalistic setting) according to the treating physician’s choice. Depressive symptom severity was graded weekly (HAMD), and serum concentrations of antidepressants were determined in weeks 3, 5, and 7 of study participation and used to adjust doses. Study details are given elsewhere (Lichter et al., 2023).

#### Munich sample

The Munich sample was collected within the MARS project (recruitment 2000–2011) (Hennings et al., 2009). A total of 1105 Caucasian participants (mean age 47.6 ± 14.1 years (range: 18–87); 53% female) were included in the naturalistic study within the first 5 days of admission as inpatients due to a depressive episode (single major depressive episode, recurrent depression, bipolar depression) in the Max Planck Institute of Psychiatry (Munich, Germany) or one of the participating clinical sites in Southern Bavaria, Germany. Diagnosis of depressive episodes was ascertained by specialized staff according to the DSM-IV criteria. Symptom severity and effectiveness of the therapy were assessed by the HAMD-21 (mean ± SD at baseline: 25.8 ± 5.9). Exclusion criteria were depressive syndromes secondary to any medical or neurological condition, acute manic, hypomanic, or mixed affective symptoms, lifetime diagnosis of alcohol dependence, illicit drug abuse, or severe medical conditions. All patients were treated independently of study participation (naturalistic setting) according to the doctor’s choice. Depressive symptom severity (HAMD) was graded weekly until treatment week 6 and afterward bi-weekly until discharge from the hospital. Therapeutic drug monitoring (TDM) was performed according to the doctor’s choice and not per protocol; serum concentrations of antidepressants were used to adjust doses. Study details are given elsewhere (Hennings et al., 2009).

Written informed consent was obtained from each participant. Both studies were approved by the local ethics committees of the Universities of Wuerzburg (104/12 and 128/15) and Munich (318/00, 21/03/2001) and carried out in accordance with the ethical principles of the Helsinki Declaration.

### Treatment response

The endpoint of the studies was week 7 (Wuerzburg sample) and week 6 (Munich sample), respectively. These time points were chosen as outcome time points for the present analysis, as an effective and consistent therapy can be expected. Treatment response was defined as ⩾50% reduction in depressive symptoms from baseline as ascertained by HAMD-21 scores (Rush et al., 2006).

### Therapeutic drug monitoring

Depending on treatment response, week 7 or 6 serum concentrations were used for analyses. TDM was applied according to the Working Group on Neuropsychopharmacology and Pharmacopsychiatry (AGNP) TDM expert group consensus guideline in both cohorts (trough levels (standardized blood withdrawal in the morning) at steady state) (Hiemke et al., 2018). Daily calibrations and internal quality control samples, integrated in each analytical series, ensure correct analytical results. The laboratories (Wuerzburg and Munich) were both certified by a quality control program (INSTAND, 2020). Consequently, the results determined in different laboratories were compatible, giving the rationale for the joint analysis of the data. TRRs were defined according to Hiemke et al. (2018).

Dimensional outliers (⩾3 SD from the mean) from serum concentrations and CD were set as missing data.

### Genotyping

Genotyping of *CYP1A2* (rs2069514, rs762551; star allele coverage, see Table 1) was performed within an assay of genotyping pharmacokinetically relevant gene variants on a MassArray Analyzer 4 system (Agena Bioscience GmbH, Hamburg, Germany) (Scherf-Clavel et al., 2022). Details are presented elsewhere (Scherf-Clavel et al., 2022). In 56 samples (45.2%), genotyping was replicated with an independent method, using the Infinium Global Screening Array-24 v1.0 BeadChip (Illumina, San Diego, CA, USA) data to ensure reliable results. SNP variants were replicated with an accuracy of 100% (rs762551), and >98% (rs2069514), respectively. Due to an update in the star allele assignment (see https://www.pharmvar.org/gene/CYP1A2; assessed: 14th of February 2025), the former *1F and *1L alleles are now reassigned as the *30.001 allele.

**Table 1.** Haplotype table giving the combination of the *CYP1A2* SNPs resulting in the respective haplotypes.

Former assignment	Updated assignment	rs2069514	rs762551
*1A	*1A	G	C
*1F	*30.001 (*1F)	G	A
*1L	*30.001 (*1F)	A	A

For matching with the Dutch Pharmacogenetics Working Group (DPWG) specifications, the *30.001 allele is named as the *1F allele throughout the manuscript. Genotype groups were determined according to the DPWG specifications into normal metabolizers (NMs), *1A/*1F genotype group, and *1F/*1F genotype group (Table 2) (Beunk et al., 2023).

**Table 2.** *CYP1A2* genotype groups were determined according to the Dutch Pharmacogenetics Working Group (DPWG) specifications.

*CYP1A2* genotype group	Definition	Diplotypes
NM	Homozygous or heterozygous for fully functional alleles	*1A/*1A
*1A/*1F genotype group	Heterozygous for a fully functional allele and allele *1F	*1A/*1F
*1F/*1F genotype group	Homozygous for the allele with increased inducibility *1F	*1F/*1F

### Statistical analyses

Statistical analyses were conducted in R v4.0.4 (R CoreTeam, 2014).

Haplotype blocks based on sample-specific linkage disequilibrium (LD) pattern from Haploview v4.1 (Barrett et al., 2005; Broad Institute, 2020) were defined for the analyzed SNPs according to gene-specific haplotype tables from the PharmVar homepage (https://www.pharmvar.org/gene/CYP1A2; assessed: 14th of February 2025). Analyses were based on haplotypes showing the highest post-probability for each individual after haplotype phasing.

Patients with *CYP1A2*-affecting comedication, as well as *CYP2D6*-, and *CYP3A4*-affecting comedication according to the Flockhart table were excluded (Flockhart et al., 2021), as these enzymes are involved in the metabolism of mirtazapine (Timmer et al., 2000).

Linear regression analyses were used to test for associations between CD and smoking status, and *CYP1A2* genotype groups, corrected for sex and age. Stepwise (forward and backward selection) model selection by Akaike information criterion (AIC) was used to find the best model for modeling the CD of mirtazapine. We did not correct for other *CYP*-enzyme variants, as evidence for an association with mirtazapine metabolism remains incomplete (PharmGKB, 2024a, 2024b; Scherf-Clavel et al., 2022; Whirl-Carrillo et al., 2021). Differences between groups (smoker/non-smoker; *CYP1A2* genotype groups) were investigated using the Mann–Whitney *U* test, and the Kruskal–Wallis test with post hoc pairwise Wilcoxon test. Chi-square test or Fisher’s exact test was performed to investigate associations between the *CYP1A2* genotype group and the serum concentration below, above, or within the TRR.

Logistic regression was applied for association analyses with treatment response as an outcome variable and serum concentration of mirtazapine, age, sex, smoking status, and *CYP1A2* genotype groups as independent variables.

A *p*-value < 0.05 was considered significant. As this was an explanatory investigation, we did not correct for multiple testing.

## Results

To increase statistical power, all analyses were restricted to the combined sample.

### Clinical and genetic characteristics

Altogether, 178 patients with TDM of mirtazapine were available. After excluding patients with CYP-affecting comedication (*N* = 18), patients with no information on smoking status (*N* = 16), patients with no information on *CYP1A2* variants (*N* = 19), and patients with CD ⩾ 3 SD from mean (*N* = 1), 124 patients were eligible for analyses.

Fifty-nine patients (47.6%) were male and 65 patients (52.4%) female. The mean age was 48.0 years (SD = 14.6, range: 18–80 years). Eighty-two patients were non-smokers and 42 were smokers. On average, 15.8 (SD = 7.9; range: 2–40) cigarettes were smoked per day. Five patients smoked 1–9 cigarettes per day, 19 patients 10–19, 14 patients 20–29, and 4 patients more than 30 cigarettes per day.

Fifteen patients were normal metabolizers (*1A/*1A; homozygous or heterozygous for fully functional alleles) on *CYP1A2*, 44 were assigned to the *1A/*1F genotype-group (*1A/*1F, heterozygous for a fully functional allele and allele *1F), and 65 patients were carriers of the *1F/*1F variant (homozygous for the allele with increased inducibility *1F).

For detailed clinical and genetic characteristics, see Table 3.

**Table 3.** Clinical and genetic characteristics of the patients.

Demographic data	*N*		Mean	SD	Min	Max
Age (years)	124		48.0	14.6	18	80
Sex (male/female)	59/65					
Non-smokers/smokers	82/42					
Number of cigarettes smoked	42		15.8	7.9	2	40
HAMD baseline	117		26.8	6.7	12	46
HAMD outcome	123		12.5	7.8	0	37
DD (Mirt) (mg)	124		43.6	22.2	7.5	120
c_p_ (Mirt) (ng/mL)	124		66.4	47.3	7	245
CD(Mirt) ((ng/mL)/(mg/day))	124		1.66	0.97	0.15	4.80
*CYP1A2*	Genotype group					
	NM		*1A/*1F	*1F/*1F		
N(*1A/*1A)	15					
N(*1A/*1F)			44			
N(*1F/*1F)				65		
Age, mean ± SD (years)	48.3 ± 18.5		45.9 ± 14.5	49.4 ± 13.9		
Sex (male/female)	9/6		21/23	29/36		
Non-smokers/smokers	9/6		28/16	45/20		
HAMD baseline, mean ± SD	22.6 ± 5.8		26.8 ± 5.7	27.7 ± 7.1		
HAMD outcome, mean ± SD	14.8 ± 7.8		11.5 ± 7.2	12.7 ± 8.1		
DD (Mirt), mean ± SD (mg)	56.5 ± 25.8		45.0 ± 22.2	39.1 ± 20.1		
c_p_ (Mirt), mean ± SD (ng/mL)	81.2 ± 51.7		70.4 ± 48.6	60.3 ± 45.1		
CD (Mirt), mean ± SD ((ng/mL)/(mg/day))	1.56 ± 0.79		1.63 ± 0.89	1.70 ± 1.06		
*CYP1A2* genotype groupSmoking status	NM		*1A/*1F		*1F/*1F	
	Smoker	Non-smoker	Smoker	Non-smoker	Smoker	Non-smoker
*N*	6	9	16	28	20	45
DD (Mirt), mean ± SD (mg)	43.8, 23.4	65, 24.9	50.6, 21.1	43.3, 22.8	43.1, 23.8	37.3, 18.3
CD (Mirt), mean ± SD ((ng/mL)/(mg/day))	1.41, 0.73	1.66, 0.86	1.22, 0.512	1.86, 0.97	1.24, 0.75	1.9, 1.12

*N*: number; SD: standard deviation; min: minimum; max: maximum; HAMD: Hamilton Depression Scale; DD: daily dose; c_p_: serum concentration; CD: dose-corrected serum concentration; NM: normal metabolizer.

### Association between serum concentration, smoking status, and *CYP1A2* variants

Linear regression analyses, including smoking status, sex, age, and *CYP1A2* genotype groups in the model, reveal a significant association between CD and smoking status (*p* = 0.002, ß = −0.53, CI (ß) = −0.86 to −0.20), sex (*p* = 0.003, ß = 0.50, CI (ß) = 0.18–0.81), and age (*p* = 0.020, ß = 0.01, CI (ß) = 0.002–0.02), with lower CD in smokers and higher CD in females and older patients. *CYP1A2* variants were not associated with CD.

Performing stepwise (forward and backward selection) model selection by AIC the best model for modeling CD of mirtazapine, accordingly, includes only age, sex, and smoking status. Thus, in the best-fitting model, smoking status, sex, and age of the patients were associated with CD of mirtazapine (smoking status: *p* = 0.002, ß = −0.53, CI (ß) = −0.86 to −0.20; sex: *p* = 0.002, ß = 0.49, CI (ß) = 0.18–0.81; age: *p* = 0.02, ß = 0.01, CI (ß) = 0.002–0.023). The model explained 20.2% of the variance in CD (*p* = 5.396 * 10^−6^).

**Figure 1.**
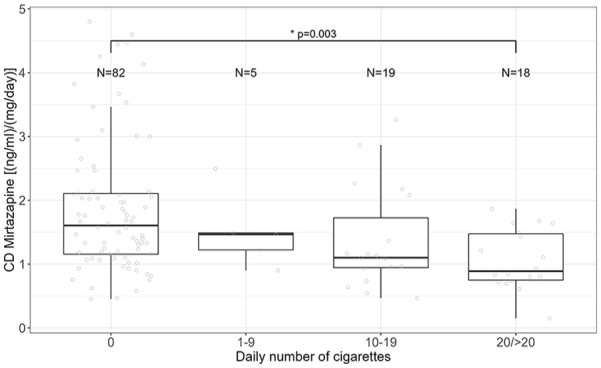
Association between CD of mirtazapine and the number of daily smoked cigarettes. Kruskal–Wallis test, *p* = 0.001; post hoc test: paired Wilcoxon test, *p* (non-smoker vs ⩾20) = 0.003).

We reran the analysis, including the number of cigarettes per day instead of smoking status. The results revealed that decreasing CD of mirtazapine was associated with patients smoking ⩾20 cigarettes per day (linear regression analysis, *p* = 0.003; ß = −0.70; CI (ß) = −1.16 to −0.25). In patients smoking 10–19 cigarettes per day, CD was not significantly lower, however, at a trend level (*p* = 0.07) (Figure 1).

### Association between serum concentration related to the TRR and *CYP1A2* variants

*N* = 72 patients showed serum concentrations within the TRR of mirtazapine (30–80 ng/mL), 21 patients below the TRR, and 31 patients above the TRR. Number of patients within/above/below the TRR did not differ between *CYP1A2* genotype groups (*x*^2^-test, *p* = 0.29). Including the smoking status of the patients did not change the result (Fisher’s exact test, *p* = 0.36).

### Inducibility of *CYP1A2* by smoking

In non-smokers (patients without *CYP1A2* induction), CD did not differ across *CYP1A2* genotype groups (Kruskal–Wallis test, *p* (non-smoker) = 0.831). This is supported by the result of linear regression analysis; accordingly, CD of patients without *CYP1A2* induction was not associated with *CYP1A2* genotype groups (*p* (*1A/*1F) = 0.72, *p* (*1F/*1F) = 0.68), corrected for sex and age. Consequently, non-smokers with different *CYP1A2* genotype groups were combined and compared to smokers (patients with *CYP1A2* inducers) assigned to different *CYP1A2* genotype groups.

**Figure 2.**
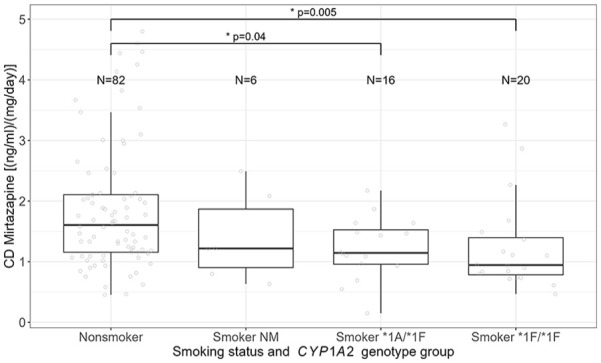
Association between CD of mirtazapine, smoking status, and *CYP1A2* genotype group.

CD of non-smokers was significantly different compared to smokers with different *CYP1A2* genotype groups (Kruskal–Wallis test, *p* = 0.004). Post hoc test revealed significantly lower CD in smokers assigned to the *1A/*1F genotype group (mean ± SD = 1.22 ± 0.51; *p* = 0.02, *p*_adj_ = 0.04), as well as smokers who were carriers of the *1F/*1F genotype (mean ± SD = 1.24 ± 0.75; *p* = 0.002, *p*_adj_ = 0.005) (Figure 2; Supplemental Figure 1) compared to non-smokers (mean ± SD = 1.86 ± 1.04). These results are supported by linear regression analysis, revealing a significant association of CD of non-smokers with CD of smokers of the *1A/*1F group (*p* = 0.04, ß = −0.50, CI (ß) = −0.98 to −0.02) and the *1F/*1F genotype (*p* = 0.007, ß = −0.61, CI (ß) = −1.04 to −0.17), but not with smokers assigned to the *CYP1A2* NM group (*p* = 0.35), corrected for sex and age.

### Association between *CYP1A2* variants and treatment response

**Figure 3.**
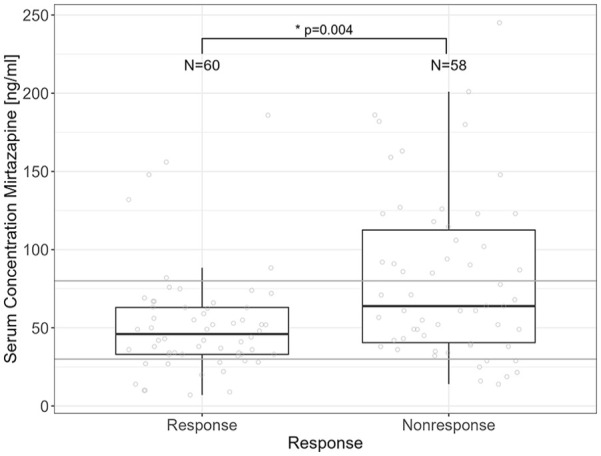
Association between serum concentration of mirtazapine and treatment response. Gray lines define the TRR of mirtazapine (30–80 ng/mL).

Considering the categorical treatment outcome (response/non-response) using logistic regression analysis, serum concentration of mirtazapine was associated with treatment response/non-response (*p* = 0.004, OR = 0.984, CI (OR) = 0.973–0.994), but not with sex, age, smoking status, and *CYP1A2* genotype group. Increasing serum concentrations were associated with increasing probability for non-response. However, the interquartile range of responders’ serum concentrations lay within the TRR, whereas the 25th to 75th quartiles of serum concentrations of non-responders includes a range above the TRR (Figure 3).

## Discussion

In the present sample, 12.1% of the patients were *CYP1A2* NM, 35.5% qualified for the *1A/*1F genotype group, and 52.4% for the *1F/*1F genotype group. This is in accordance with data from the Royal Dutch Pharmacist’s Association (KNMP) in a reference population from the Netherlands (12.0% NM, 45.0% *1A/*1F, 43.0% *1F/*1F) (Royal Dutch Pharmacist’s Association, 2021) (two-tailed *z*-test; NM, *p* = 1.0; *1A/*1F, *p* = 0.22; *1F/*1F, *p* = 0.23).

CD of mirtazapine was associated with smoking status, sex, and age, but not with *CYP1A2* genotype groups. This is in accordance with previous studies showing an association of CD with smoking status of the patients (Lind et al., 2009; Oliveira et al., 2017; Scherf-Clavel et al., 2019). Despite the evidence that there is an association of mirtazapine serum concentration with smoking, this, to the best of our knowledge, is the first study to include *CYP1A2* genotype groups in regression analysis. Serum concentration related to the TRR was also not associated with *CYP1A2* variants. However, TDM was used for dose optimization; therefore, serum concentrations of the patients were titrated to the TRR; consequently, we did not expect an association of genetic variants with serum concentrations related to the TRR. To summarize, we did not show an association of CD or serum concentrations related to the TRR with *CYP1A2* genotype groups; however, we observed differences in CD of mirtazapine between non-smokers and smokers who were carriers of the different genetic variants. CD of non-smokers and *CYP1A2* NM smokers did not differ, but CYP1A2*1A/*1F and *1F/*1F smokers showed 34.4% and 33.4% lower CD compared to non-smokers, respectively. Thus, mirtazapine serum concentration in smokers who are CYP1A2*1F carriers is lower compared to non-carriers of this gene variant, due to higher inducibility of CYP1A2*1F (Beunk et al., 2023; Royal Dutch Pharmacist’s Association, 2021). Consequently, in smokers carrying the *1F variant serum concentration of mirtazapine will increase if the patient stops smoking or switches to e-cigarettes, as not the tobacco, but the polycyclic aromatic hydrocarbons in cigarette smoke are responsible for *CYP1A2* induction (Al-Arifi et al., 2012; Berm et al., 2015; Kroon, 2007). To give an example, in a carrier of the *1F variant, receiving a daily dose of 30 mg mirtazapine serum concentration may increase from 37 to 56 ng/mL, which may significantly affect effectiveness and tolerability, although existing evidence on both relating to CD is lacking. However, in combination with other psychopharmacological treatment interventions, which are frequently needed in severely affected patients, this unexpected increase in mirtazapine CD due to smoking cessation might induce a potentiated negative impact. Without considering genetics, after smoking cessation, the individual change in *CYP1A2* activity was reported to range from a 1.0-fold to a 7.3-fold decrease in activity (Dobrinas et al., 2011). Thus, especially in CYP1A2*1F carriers, physicians should alert the patient about a possible unplanned increase/decrease in serum concentration of mirtazapine when changing smoking habits, and therefore the risk of adverse drug effects or worsening of therapy response. Nevertheless, as the group of non-smoking NM was small (*N* = 6), it is not clear whether these results will remain the same with a larger number of patients. For clinical practice, we, therefore, suggest focusing on smoking status rather than on *CYP1A2* genetics during mirtazapine treatment.

It was reported that not only smoking status but also the number of cigarettes impacts *CYP1A2* activity (Dobrinas et al., 2011). *CYP1A2* activity was found to increase with the number of cigarettes smoked per day, with major differences found between groups smoking 10–19 and 30–39, or 40–70 cigarettes per day (Dobrinas et al., 2011). In our sample, 5 patients smoked 1–9 cigarettes per day, 19 patients smoked 10–19, and 18 patients smoked 20 or more cigarettes per day. Corroborating previous results, we found a significantly decreasing CD of mirtazapine in patients smoking ⩾20 cigarettes per day compared to non-smokers. However, in patients smoking 10–19 cigarettes per day, CD was also lower but on trend level (*p* = 0.07). While this supports previous findings, this may also indicate that the smoking effects on *CYP1A2* might not be linear. Therefore, a bigger sample size might be needed to depict those effects in the intermediate group of 10–19 cigarettes per day. In addition, other influencing factors affecting the CD of mirtazapine could partly explain our results, other metabolizing enzymes, for example other metabolizing enzymes *CYP2D6* and *CYP3A4* (Stormer et al., 2000). Analysis on inducibility of *CYP1A2* in association with the number of cigarettes and genotype group was impossible due to small sample sizes.

*CYP1A2* genotype groups were not associated with treatment outcome; however, serum concentration of mirtazapine was different between response outcomes. In our samples, increasing serum concentrations were associated with increasing probability for non-response. The interquartile range of responders’ serum concentrations lay within the TRR, whereas the 25th to 75th quartiles of serum concentrations of non-responders include a range above the TRR (Figure 3). Thus, serum concentrations within the TRR were adequate for treatment response, and patients did not benefit from serum concentrations above the TRR. Mainly, clinicians would decide to increase the dosage in non-response conditions. This means that the dosage was increased primarily due to inadequate response to pharmacological treatment to achieve beneficial therapeutic effects. In addition, but less likely, as adverse drug effects were not recorded systematically, the experienced side effects under elevated serum concentrations may be more prominent than the therapeutic effect and, in consequence, may have been interpreted as nonresponse. In consequence, if patients do not respond to mirtazapine serum concentration within the TRR, further increase in the dosage seems not to be beneficial, and a switch to another medication should be considered to increase the probability of treatment response. In our clinical cohorts, the mean daily dose of mirtazapine was 43.6 mg, which is in the upper range of the suggested dosing. Due to our inpatient setting, at baseline, the mean HAMD of patients receiving mirtazapine was 27, indicative of a severe depressive syndrome. We assume that the severity of the depressive symptoms was the main reason for the dosage ranges to achieve an adequate clinical response. Nevertheless, in both studies, dosing was adjusted by involvement of TDM; thus, the physicians increased dosages with monitoring of the serum concentration to prevent adverse effects.

### Strengths and limitations

The present combined data from two independent observational studies provide real-world data from a naturalistic setting, and, therefore, the results are relevant for clinical routine. However, our design also had some limitations: Only Caucasians were included in both studies of the present analyses; therefore, our results are only valid for Caucasians. Serum concentrations were measured in two independent laboratories; however, both were certified by a quality control program (INSTAND, 2020). Thus, the results determined in different laboratories were compatible, giving the rationale for the joint analysis of the data. The physicians were not aware of post hoc PGx data, but were aware of TDM results; therefore, dosing was adjusted to the serum concentrations of the drugs. Adverse drug effects were not systematically reported. Moreover, we did not adjust for caffeine consumption, even if caffeine may inhibit *CYP1A2* (Hiemke et al., 2024). Despite the large number of patients enrolled in both samples (GEParD, MARS), only a limited number of patients were eligible for the analyses of mirtazapine serum concentration and *CYP1A2* genetics, due to the naturalistic setting of the treatment; thus, it is even more impressive that an association of mirtazapine serum concentration, smoking status, and *CYP1A2* gene variation was reported. However, especially the group of non-smoking NM was limited (*N* = 6); consequently, it is not clear if these results will remain the same with a larger number of patients.

## Conclusion

Only 12.1% of the patients were assigned to the *CYP1A2* NM phenotype, whereas 35.5% were assigned to the *1A/*1F genotype group, and 52.4% to the *1F/*1F genotype group, with CYP1A2*1F being described as a highly inducible variant. Smokers, assigned to the CYP1A2*1A/*1F and *1F/*1F genotype groups, showed 34.4% and 33.4% lower mirtazapine CD compared to non-smokers. CD of mirtazapine in *CYP1A2* NM smokers did not differ from non-smokers. In consequence, the present results suggest that in CYP1A2*1F carriers who quit smoking or switch to e-cigarettes, physicians should be sensitized to monitor an increase in side effects or intolerance due to the expected increase in mirtazapine serum concentrations. In these cases, measurement of mirtazapine CD and/or subsequent dosage reduction is recommended. However, as the sample size was limited for non-smoking NM (*N* = 6), and therefore the benefit of prior genotyping of CYP1A2*1F cannot be definitely derived for clinical practice, our results show the relevance of monitoring the smoking status in patients treated with mirtazapine in the first place. In consequence, the clinical relevance of *CYP1A2* genotyping prior to treatment with drugs metabolized by *CYP1A2* needs further investigation. We are aware that the results are based on a restricted sample size; however, since such real-life data are rare, the results might inform further developments in precision medicine in psychiatry, as well as routine clinical practice.

## Supplemental Material

sj-docx-1-jop-10.1177_02698811251337387 – Supplemental material for CYP1A2 genotype-dependent effects of smoking on mirtazapine serum concentrations Supplemental material, sj-docx-1-jop-10.1177_02698811251337387 for CYP1A2 genotype-dependent effects of smoking on mirtazapine serum concentrations by Maike Scherf-Clavel, Heike Weber, Carolin Weiß, Catherina Klüpfel, Saskia Stonawski, Leif Hommers, Stefan Unterecker, Katharina Domschke, Andreas Menke, Sarah Kittel-Schneider, Sebastian Walther, Jürgen Deckert and Angelika Erhardt-Lehmann in Journal of Psychopharmacology

## References

[bibr1-02698811251337387] Al-Arifi MN Maayah ZH Alshamrani AA , et al. (2012) Impact of cigarette smoke exposure on the expression of cardiac hypertrophic genes, cytochrome P450 enzymes, and oxidative stress markers in rats. J Toxicol Sci 37: 1083–1090. 23038017 10.2131/jts.37.1083

[bibr2-02698811251337387] Barrett JC Fry B Maller J , et al. (2005) Haploview: Analysis and visualization of LD and haplotype maps. Bioinformatics 21: 263–265. 15297300 10.1093/bioinformatics/bth457

[bibr3-02698811251337387] Berm EJJL Ruijsbroek R Loonen AJM , et al. (2015) [Switching to e-cigarettes affects drug concentration]. Ned Tijdschr Geneeskd 159: A9090. 26200424

[bibr4-02698811251337387] Beunk L Nijenhuis M Soree B , et al. (2023) Dutch Pharmacogenetics Working Group (DPWG) guideline for the gene-drug interaction between *CYP2D6, CYP3A4* and *CYP1A2* and antipsychotics. Eur J Hum Genet 32(3): 278–285. 37002327 10.1038/s41431-023-01347-3 PMC10923774

[bibr5-02698811251337387] Breslau N Kilbey M Andreski P (1991) Nicotine dependence, major depression, and anxiety in young adults. Arch Gen Psychiatry 48: 1069–1074. 1845224 10.1001/archpsyc.1991.01810360033005

[bibr6-02698811251337387] Broad Institute (2020) Haploview. Available at: https://www.broadinstitute.org/haploview/haploview (accessed 10 January 2020).

[bibr7-02698811251337387] Brown RA Lewinsohn PM Seeley JR , et al. (1996) Cigarette smoking, major depression, and other psychiatric disorders among adolescents. J Am Acad Child Adolesc Psychiatry 35: 1602–1610. 8973066 10.1097/00004583-199612000-00011

[bibr8-02698811251337387] Chaiton MO Cohen JE O’Loughlin J , et al. (2009) A systematic review of longitudinal studies on the association between depression and smoking in adolescents. BMC Public Health 9: 356. 19772635 10.1186/1471-2458-9-356 PMC2758872

[bibr9-02698811251337387] Dobrinas M Cornuz J Oneda B , et al. (2011) Impact of smoking, smoking cessation, and genetic polymorphisms on *CYP1A2* activity and inducibility. Clin Pharmacol Ther 90: 117–125. 21593735 10.1038/clpt.2011.70

[bibr10-02698811251337387] Flockhart D Thacker D McDonald C , et al. (2021) The Flockhart cytochrome P450 drug-drug interaction table. Available at: https://medicine.iu.edu/internal-medicine/specialties/clinical-pharmacology/drug-interaction-flockhart-table (accessed 2 February 2021).

[bibr11-02698811251337387] Hennings JM Owashi T Binder EB , et al. (2009) Clinical characteristics and treatment outcome in a representative sample of depressed inpatients—findings from the Munich Antidepressant Response Signature (MARS) project. J Psychiatr Res 43: 215–229. 18586274 10.1016/j.jpsychires.2008.05.002

[bibr12-02698811251337387] Hiemke C Bergemann N Clement HW , et al. (2018) Consensus guidelines for therapeutic drug monitoring in neuropsychopharmacology: Update 2017. Pharmacopsychiatry 51: 9–62. 28910830 10.1055/s-0043-116492

[bibr13-02698811251337387] Hiemke C Haen E Eckermann G , et al. (2024) PSIAC. Available at: https://www.psiac.de/ (accessed 03 August 2023).

[bibr14-02698811251337387] INSTAND (2020) Available at: https://www.instand-ev.de/ueber-instand-ev/instand-ev.html (accessed 10 February 2020).

[bibr15-02698811251337387] Jaquenoud Sirot E Harenberg S Vandel P , et al. (2012) Multicenter study on the clinical effectiveness, pharmacokinetics, and pharmacogenetics of mirtazapine in depression. J Clin Psychopharmacol 32: 622–629. 22926595 10.1097/JCP.0b013e3182664d98

[bibr16-02698811251337387] Kroon LA (2007) Drug interactions with smoking. Am J Health Syst Pharm 64: 1917–1921. 17823102 10.2146/ajhp060414

[bibr17-02698811251337387] Laika B Leucht S Heres S , et al. (2010) Pharmacogenetics and olanzapine treatment: *CYP1A2**1F and serotonergic polymorphisms influence therapeutic outcome. Pharmacogenomics J 10: 20–29. 19636338 10.1038/tpj.2009.32

[bibr18-02698811251337387] Lichter K Klüpfel C Stonawski S , et al. (2023) Deep phenotyping as a contribution to personalized depression therapy: The GEParD and DaCFail protocols. J Neural Trans 130: 707–722. 10.1007/s00702-023-02615-8 PMC10121520 36959471

[bibr19-02698811251337387] Lind AB Reis M Bengtsson F , et al. (2009) Steady-state concentrations of mirtazapine, N-desmethylmirtazapine, 8-hydroxymirtazapine and their enantiomers in relation to cytochrome P450 2D6 genotype, age and smoking behaviour. Clin Pharmacokinet 48: 63–70. 19071885 10.2165/0003088-200948010-00005

[bibr20-02698811251337387] Oliveira P Ribeiro J Donato H , et al. (2017) Smoking and antidepressants pharmacokinetics: A systematic review. Ann Gen Psychiatry 16: 17. 28286537 10.1186/s12991-017-0140-8 PMC5340025

[bibr21-02698811251337387] PharmGKB (2024a) Guideline annotations. Available at: https://www.pharmgkb.org/guidelineAnnotations (accessed 15 March 2024).

[bibr22-02698811251337387] PharmGKB (2024b) Mirtazapine—clinical annotations. Available at: https://www.pharmgkb.org/chemical/PA450522/clinicalAnnotation (accessed 15 March 2024).

[bibr23-02698811251337387] R CoreTeam (2014) R: A language and environment for statistical computing. R Core Team. Available at: https://www.R-project.org/

[bibr24-02698811251337387] Royal Dutch Pharmacist’s Association (2021) General Background Text Pharmacogenetics—CYP1A2. The Netherlands: Royal Dutch Pharmacist’s Association.

[bibr25-02698811251337387] Rush AJ Kraemer HC Sackeim HA , et al. (2006) Report by the ACNP Task Force on response and remission in major depressive disorder. Neuropsychopharmacology 31: 1841–1853. 16794566 10.1038/sj.npp.1301131

[bibr26-02698811251337387] Sachse C Brockmöller J Bauer S , et al. (1999) Functional significance of a C–>A polymorphism in intron 1 of the cytochrome P450 *CYP1A2* gene tested with caffeine. Br J Clin Pharmacol 47: 445–449. 10233211 10.1046/j.1365-2125.1999.00898.x PMC2014233

[bibr27-02698811251337387] Sangüesa E Cirujeda C Concha J , et al. (2022) Exploring the usefulness of plasma level determination and pharmacogenetics for patients treated with clozapine. Per Med 19: 181–192. 35259926 10.2217/pme-2021-0029

[bibr28-02698811251337387] Scherf-Clavel M Samanski L Hommers LG , et al. (2019) Analysis of smoking behavior on the pharmacokinetics of antidepressants and antipsychotics: Evidence for the role of alternative pathways apart from CYP1A2. Int Clin Psychopharmacol 34: 93–100. 30557209 10.1097/YIC.0000000000000250

[bibr29-02698811251337387] Scherf-Clavel M Weber H Wurst C , et al. (2022) Effects of pharmacokinetic gene variation on therapeutic drug levels and antidepressant treatment response. Pharmacopsychiatry 55: 246–254. 35839823 10.1055/a-1872-0613 PMC9458342

[bibr30-02698811251337387] Seifert J Bleich S Seifert R (2022) Depression, angststörungen, bipolare störung, schizophrenie, aufmerksamkeitsdefizithyperaktivitätssyndrom. In: W-D Ludwig B Mühlbauer R Seifert (eds) Arzneiverordnungs-Report 2022. Berlin, Heidelberg: Springer Berlin Heidelberg, pp. 451–495.

[bibr31-02698811251337387] Stormer E von Moltke LL Shader RI , et al. (2000) Metabolism of the antidepressant mirtazapine in vitro: contribution of cytochromes P-450 1A2, 2D6, and 3A4. Drug Metab Dispos 28: 1168–1175. 10997935

[bibr32-02698811251337387] Timmer CJ Sitsen JM Delbressine LP (2000) Clinical pharmacokinetics of mirtazapine. Clin Pharmacokinet 38: 461–474. 10885584 10.2165/00003088-200038060-00001

[bibr33-02698811251337387] van der Weide J Steijns LS van Weelden MJ (2003) The effect of smoking and cytochrome P450 *CYP1A2* genetic polymorphism on clozapine clearance and dose requirement. Pharmacogenetics 13: 169–172. 12618594 10.1097/00008571-200303000-00006

[bibr34-02698811251337387] Whirl-Carrillo M Huddart R Gong L , et al. (2021) An evidence-based framework for evaluating pharmacogenomics knowledge for personalized medicine. Clin Pharmacol Ther 110: 563–572. 34216021 10.1002/cpt.2350 PMC8457105

[bibr35-02698811251337387] Ziedonis D Hitsman B Beckham JC , et al. (2008) Tobacco use and cessation in psychiatric disorders: National Institute of Mental Health report. Nicotine Tob Res 10: 1691–1715. 19023823 10.1080/14622200802443569

