# Supplementary material for: CYP1A2 genotype-dependent effects of smoking on mirtazapine serum concentrations
Source: J Psychopharmacol. 2025 May 12;39(8):847–54. doi: 10.1177/02698811251337387 (PMC12287552; doi:10.1177/02698811251337387)
Supplement: sj-docx-1-jop-10.1177_02698811251337387 – Supplemental material for CYP1A2 genotype-dependent effects of smoking on mirtazapine serum concentrations [file sj-docx-1-jop-10.1177_02698811251337387.docx]

**Supplemental Figure 1**

**to:**

CYP1A2 genotype dependent effects of smoking on mirtazapine serum concentrations


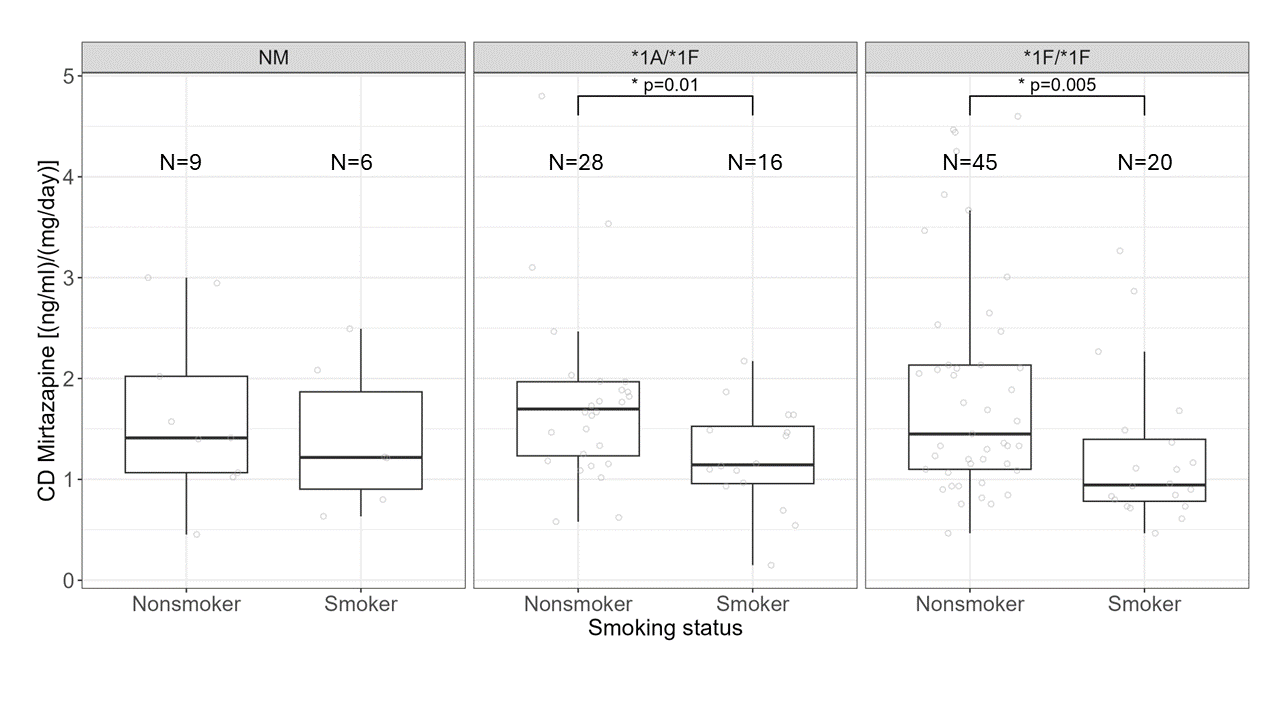


Supplemental Figure 1 CD of mirtazapine was associated with smoking status in carrier of the *1A/*1F, as well as *1F/*1F genotype group.
